# Clemastine Enhances Myelination, Delays Axonal Loss and Promotes Functional Recovery in Spinal Cord Injury

**DOI:** 10.1007/s11064-021-03465-0

**Published:** 2021-10-18

**Authors:** Weihong Du, Yongbing Deng, Rong Jiang, Luyao Tong, Ruixue Li, Xue Jiang

**Affiliations:** 1 Department of Biochemistry and Molecular Biology, Molecular Medicine and Cancer Research Center, School of Basic Medicine, Chongqing Medical University, Chongqing, 400016 China; 2 Department of Chongqing Emergency Medical Center, Chongqing University Center Hospital, School of Medicine, Chongqing University, Chongqing, 400014 China; 3 Department of Histology and Embryology, Chongqing Key Laboratory of Neurobiology, Third Military Medical University, Chongqing, 400038 China

**Keywords:** Spinal cord injury, Myelination, Axon, Clemastine, Oligodendrocytes

## Abstract

Recent evidence has shown that demyelination occurs along with axonal degeneration in spinal cord injury (SCI) during the secondary injury phase. Oligodendrocyte precursor cells (OPC) are present in the lesions but fail to differentiate into mature oligodendrocytes and form new myelin. Given the limited recovery of neuronal functions after SCI in adults without effective treatment available so far, it remains unknown whether enhancing OPC differentiation and myelination could benefit the recovery of SCI. To show the significance of myelin regeneration after SCI, the injury was treated with clemastine in the rat model. Clemastine is an FDA-approved drug that is potent in promoting oligodendrocyte differentiation and myelination in vivo, for four weeks following SCI. Motor function was assessed using sloping boards and grid walking tests and scored according to the Basso, Beattie, and Bresnahan protocol. The myelin integrity and protein expression were evaluated using transmission electron microscopy and immunofluorescence, respectively. The results indicated that clemastine treatment preserves myelin integrity, decreases loss of axons and improves functional recovery in the rat SCI model. The presented data suggest that myelination-enhancing strategies may serve as a potential therapeutic approach for the functional recovery in SCI.

## Introduction

Spinal cord injury (SCI) is a violent trauma with high occurrence rate in events such as car accidents and falls that typically leads to sustained motor, autonomic and sensory dysfunction. The incidence of SCI is rising globally each year; in China in particular, it has been estimated to affect between five and ten individuals per million [1]. SCI is divided into primary injury and secondary injury. Primary injury is the initial mechanical injury caused by local tissue deformation and trauma. Secondary injury involves oligodendrocyte death and axon demyelination, which may cause further axonal degeneration [2]. The clinical treatment of SCI includes methylprednisolone, gangliosides and surgical intervention [3]. The clinical treatment guidelines of SCI point out the importance of spinal cord decompression, spinal stability restoration, and cardiopulmonary and metabolic function support [4]. However, therapies that can promote functional recovery following SCI remain currently unavailable.

Myelin sheaths generated by oligodendrocytes in the central nervous system (CNS) insulate axons and provide metabolic support. Demyelinated axons are more vulnerable to detrimental microenvironments, thus, promoting remyelination can protect axons against degeneration [5]. Recent evidence has also shown a potential reason for demyelination in SCI [6] is due to oligodendrocyte (OL) apoptosis [7–10]. Given that myelin integrity is important for axon survival and function [11], this paper proposes that remyelination is beneficial for the recovery of neurofunctions following SCI [12–14]. Complete remyelination is possible as oligodendrocyte precursor cells (OPCs) exist abundantly in the CNS and maintain their abilities to differentiate and form new myelin [15]. However, it is noted that OPC differentiation is arrested in the lesions, resulting in a failure of remyelination [16].

Recent advances in high-throughput screening have identified a group of muscarinic antagonists that can promote the differentiation and myelination of oligodendrocytes; Among them, clemastine is an FDA-approved drug that can efficiently pass the blood–brain barrier [17]. Clemastine has been shown to be effective in promoting myelination in hypoxic mouse brains and demyelinating mouse models [18–21]. The SCI model in this paper mimics partial damage of the spinal cord, without very severe disruptions. The SCI model was treated with clemastine and the effect on myelin integrity and neuronal functions was examined. The results suggest that demyelination can lead to axonal degeneration and prolongation of functional impairment, and that a cleamstine treatment strategy is a promising method for functional recovery, following SCI.

## Materials and Methods

### Animals

A total of 60 specific-pathogen-free 8-week-old adult female Sprague–Dawley rats weighing 200–240 g were purchased from the Laboratory Animal Center of Chongqing Medicine University in China (license No. SYXK (Su) 2018–0003). The animals were housed individually and given adequate water and food. The living conditions of the rats included a temperature of 25 °C and a 12-h light/dark cycle.

### Experimental Design

To explore the effect of clemastine on SCI, the rats were randomized to receive either SCI or sham surgery, followed by randomization to treatment with either clemastine or 0.9% saline vehicle control, as described below. Group assignments were coded according to best practice guidelines, to ensure that investigators were not informed during data collection and analysis.

### Spinal Cord Injury Model

(1) The rats fasted for 12 h before surgery and were anesthetized with isoflurane. (2) The animals were fixed, a shaver was used to remove the hair on their back, and the dorsal area was disinfected. (3) The T10 position was approached according to the rule of the most prominent T2 thoracic spinous process. (4) An incision of about 4 cm in length was made on the skin, centering on the T10 position, the mesangium was cut with ophthalmic scissors, and the silvery white aponeurosis was exposed. The disappeared part was the T10 spinal cord, and at the same time, the first place L1 of the silver-white tendon cross was used as an auxiliary location. (5) The tendon was cut with ophthalmic scissors to expose the spinous process and spine, the lamina was cut with dead skin scissors, and the upper spine was clamped with hemostatic forceps for fixation. Micro forceps and dead skin scissors were used to open the lamina, taking care not to injure the spinal cord tissue. (6) Arterial clamps were used to clamp the spinal cord T10 tissue, compress the spinal cord laterally to a thickness of 0.35 mm, and maintain it for 15 s. A twitch in the hind limbs and tail signaled a successful surgery. Sham-operated rats were subjected to the same surgical operation, except for spinal cord compression. (7) In order to complete the operation, the injured area was sutured, the animal was removed from the anesthesia, recovered in the heating room for ~ 1 h under close observation, and returned to the group shelter.

### Drug Treatment

Clemastine (Cat #: S1847; SelleckChem, Houston, TX) was dissolved in DMSO at 35 mg/mL before further dilution in 0.9% NaCl. The final concentration of DMSO was 0.003 v/v. The rats were treated daily with either clemastine at 10 mg/kg (SCI group) or equivalent vehicle solvent (control group) from P0 to P28 via way of gavage. The rat model of SCI was treated starting from three hours after injury.

### Behavioral Testing

1. Basso, Beattie, and Bresnahan (BBB) locomotor behavior score: The rats were placed in the testing field to familiarize themselves with the environment before surgery; the BBB score was initiated on the first day after surgery, and the BBB score test was observed and scored immediately for 4 min.
2. Grid walking analysis: The rats were required to pass through a horizontal wire mesh grid (mesh area 2.5 × 2.5 cm^2^), by walking within the grid for at least 30 s within 3 min of initiation of the test. A hindlimb that is completely detached from the grid was considered an error. The total number of lower limb movements recorded in the specified time and the total number of errors in the lower limbs were compared.
3. Inclined plate experiment: The overall evaluation of the muscle strength of the rat limbs was evaluated in this experiment. The swash plate surface pad was placed with a 6 mm thick rubber pad. The rat was placed in a direction perpendicular to the longitudinal axis of the slant plate, and the angle between the swash plate and the horizontal plane was gradually increased until the rat stayed on the plate for no more than 5 s. This angle was measured three times in each session and was then averaged.

### Electrophysiological Test

Detection of motor evoked potential: The stimulating electrode was placed 2 mm in front of the coronal suture and 2 mm below the midline, and the recording electrode was placed in the middle of the anterior tibial muscle group. The reference electrode was placed 1 cm distally to the recording electrode on the same side, and tightly attached to the tail with 0.9% NaCl, while the unilateral stimulation intensity was 4 V. The instrument employed was the BL-420F biological function experimental system of China Taimeng Company. Each group of consisted of ten animals, and each animal was tested three times.

### Tissue Processing

The rats were deeply anesthetized with 1% pentobarbital, then, the blood was flushed with 0.9% normal saline at 37 °C, 4% paraformaldehyde in 0.1 M PB was perfused into the heart, and the spinal cord around the injury site was removed and fixed overnight in 0.1 M PB with 4% paraformaldehyde. The spinal cord was then subjected to gradient dehydration in 20% and 30% sucrose and embedded in the compound of the optimal cutting temperature (OCT Compound, SAKURA, 4583, USA) on a cryostat (MS 1850, Leica, Wetzlar, Germany), and the coronal spinal cord tissue was sectioned at a thickness of 20 microns.

### Luxol Fast Blue Staining

Preheating of 0.1% Luxol Fast Blue solution (LFB) was performed, and samples were placed into a 60 °C oven for 2 h. The paraffin sections were placed in PBS for 3 min, deionized water for 3 min, 70%, 80%, 90%, 95% and absolute ethanol for 3 min each, and then quickly placed into the preheater before being placed in the warmed LFB dye solution for 4 h. Subsequently, the samples were removed from the LFB solution and allowed to cool at room temperature. They were then placed into 95% ethanol and deionized water for 3 min, respectively. The film was placed in differentiation solution for 30 s and 70% ethanol for 45 s. The differentiation process was observed under a microscope until the gray matter and white matter boundaries were clear. Finally, the samples were dehydrated and mounted. The myelinated area was quantified with Image-Pro Plus professional software.

### Immunofluorescence Staining

Frozen sections from five rats randomly selected from each group were prepared, each animal sample was divided into five consecutive equidistant sections according to the order of the spinal cord, and samples from each group were randomly selected for immunofluorescence staining. For immunofluorescence staining, the floating section was sealed with 5% bovine serum albumin (BSA) and 0.2% Triton X-100, incubated at 37 °C for 1 h, and then incubated with the primary antibody overnight at 4 °C. The primary antibody was washed with PBS. Then, the fluorescent secondary antibody was incubated at room temperature for 2 h, and then washed with PBS. Finally, the film was covered. Primary antibodies included rat anti-MBP (1:500, Millipore, Cat: MAB395), mouse anti-CC1 (1:500, Calbiochem, Cat: OP80), rabbit anti-NG2 (1:1000, Millipore, Cat: MAB5320), and rabbit anti-NF200 (1:1000, Sigma-Aldrich, Cat: N4142). Secondary antibodies included the following: AlexaFluor-488- and AlexaFluor-568-, conjugated secondary antibodies against rabbit, mouse, or rat (1:1000; Invitrogen). Nuclei were counterstained with DAPI. Fluorescent images were captured using a confocal laser-scanning microscope (Olympus, FV1000, Shinjuku, Tokyo).

### Electron Microscopy

Spinal cord tissue was prefixed with 3% glutaraldehyde, and then fixed with 1% osmium tetroxide. Dehydration was performed step-by-step with acetone. The dehydrated sample was passed through a dehydrating agent and epoxy resin (Epon812) penetrating solution successively, with ratios of 3:1, 1:1, and 1:3, for 30–60 min each. The infiltrated sample block was placed into a suitable mold, filled with embedding liquid. A solid matrix was formed after heating and polymerization. Ultrathin sections with a thickness of approximately 50 nm were prepared using an ultrathin microtome (EM KMR3), and were stained with uranyl acetate for 10–15 min, and then with lead citrate for 1–2 min, at room temperature. Transmission electron microscopy (JEM- 1400 PLUS) was used for observation.

### Quantification and Statistical Analysis

#### Quantification

Counting method of NG2 + , CC1 + , CD3, Iba1, SMI32 and NF200 + cells or axons per unit area of spinal cord white matter area: Take a photo with a fluorescence microscope at 400 × magnification. Each animal had 3–5 slices, and 5–7 fields in the white matter area of the spinal cord were selected from each slice. The number of positive cells or axons was calculated in each field, then the number of positive cells or axons was counted per unit area. All quantification was performed using Image-Pro Plus 5.0 (Media Cybernetics, Silver Spring, MD, USA). The person who performed the quantification was blinded to genotype.

Quantitative method of MBP immunofluorescence intensity: A photo of the white matter area of the spinal cord was taken with a fluorescence microscope at 400 × magnification. At least 25 fields of view for the white matter area of the spinal cord were selected from each animal, and then Image-Pro Plus software 5.0 (Media Cybernetics, Silver Spring, MD, USA) was used.

### Statistical Analysis

Data are presented as means ± SEM. An unpaired t-test was used to determine the significance between two experimental groups. When the original data showed a non-normal distribution, the non-parametric Mann–Whitney U test was used. A significant difference was indicated as *p < 0.05, **p < 0.01 or ***p < 0.001.

## Results

### Clemastine Treatment Improves Neurobehavioral Recovery after Compressed Spinal Cord Injury in Rats

**Fig. 1:**
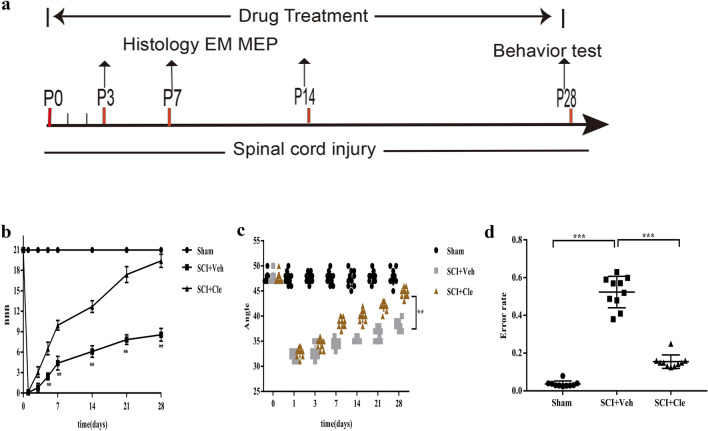
Clemastine promotes locomotor function recovery after SCI in rats. **a** Schematic diagram showing the time course of clemastine treatment and the situation of the tissue. **b** BBB (Basso, Beattie, and Bresnahan) scores. Clemastine treatment (SCI + Cle) provided the greatest improvements in BBB scores compared to the vehicle-treated groups (SCI + Veh) from one to twenty-eight days postinjury. **c** Slope experiments. The angle of the clemastine-treated groups maintained for 5 s was obviously higher than the angle of the vehicle-treated group from one to twenty-eight days postinjury. **d** Gridwalk analysis. The pace of the hind leg error rate revealed sensory-motor recovery in the SCI + Cle group at twenty-eight days postinjury compared to the vehicle-treated group. [n = 10 for the sham group, n = 10 for the SCI + Veh group, n = 10 for the SCI + Cle group]. Data are expressed as the mean ± SEM. *p < 0.05, **p < 0.01, and ***p < 0.001

To test the effects of clemastine on motor behavior in SCI, the rats were treated with clemastine at 10 mg/kg per day during SCI and the motor function was scored according to the BBB locomotor scale. The BBB scoring value is positively correlated to the degree of damage in SCI and can characterize hindlimb motor function. One-way ANOVA revealed significant improvements in the BBB score in clemastine-treated group of rats, compared to the vehicle-treated group (Fig. 1B). At 4 weeks, the vehicle-treated rats reached an average score of 8.5 ± 0.966. In contrast, the clemastine-treated group reached an average score of 19.4 ± 0.956, showing significant functional improvement (p < 0.001). Similarly, a slope experiment was also performed to supplement the BBB score, and the same result was observed (Fig. 1C). A grid walking analysis experiment was also conducted at 4 weeks to observe the recovery of fine motor regulation of the rat legs after injury. Grid walk analysis revealed that sensory and motor function showed an overall improvement after 4 weeks of SCI in both clemastine-treated group and vehicle-treated group (Fig. 1D). At 4 weeks after SCI, the average footfall rate in the clemastine-treated group was 0.1547 ± 0.0356% compared to the 0.5238 ± 0.0833% footfall rate in the vehicle-treated group. The clemastine-treated group showed a trend of reduced footfalls, which reached significance (p < 0.001).

These results indicate that clemastine treatment is capable to improve motor function recovery in rat models of SCI.

### Clemastine Promotes Motor Conduction after Spinal Cord Injury

**Fig. 2:**
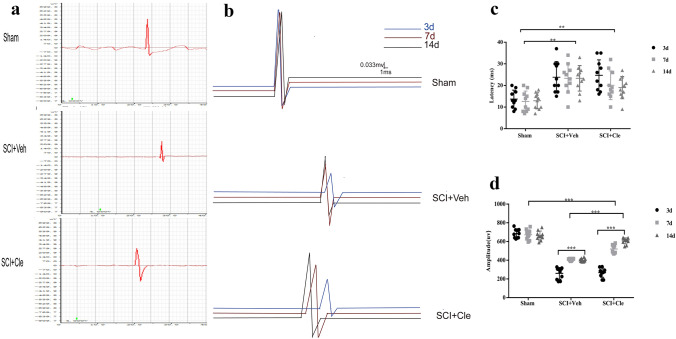
Time course of the effects of clemastine treatment on the latency and amplitude of MEPs. **a** Primary data of each group of MEP on the fourteenth day of spinal cord injury. **b** The change trend of MEP at different time points in different groups. **c** The peak amplitude was 607.14 ± 32.51 μV (n = 10, p = 0.004) in the clemastine-treated group, while those treated with vehicle had peak amplitudes of 401.04 µm. **d** The average latency in the clemastine-treated group was 19 ± 5.16 ms compared to 23.3 ± 5.89 ms in the vehicle-treated group. The clemastine-treated group showed a trend of reduced latencies, which failed to reach significance (n = 10, p = 0.1)

Motor evoked potential (MEP) signals are transmitted along the anterior lateral cord of the spinal cord, and are more sensitive to experimental SCI, consistent with motor conduction. The restoration of MEP precedes animal motor function and is useful for the purpose of diagnosing SCI and prognosis. Motor function of the spinal cord is accurately and comprehensively reflected in MEP signals. Compared with the vehicle-treated group, the MEP amplitude was significantly greater in the clemastine-treated group (Fig. 2A, B). In particular, the average peak amplitude was 607.14 ± 32.51 μV and 401.04 μV in the clemastine-treated group and vehicle-treated group, respectively (p = 0.004) (Fig. 2B). The average latency in the clemastine-treated group was 19 ± 5.16 ms, compared with 23.3 ± 5.89 ms in the vehicle-treated group. The clemastine-treated group showed a trend of reduced latency, which failed to reach significance (p = 0.1). Peak latencies were not significantly different among groups (Fig. 2C). Altogether, MEP results showed that after SCI, clemastine may contribute to prevent the degeneration of axons and promote the overall synchronization and integrity of the conduction pathway.

### Clemastine Enhances Myelination after Compressed Spinal Cord Injury in Rats

**Fig. 3:**
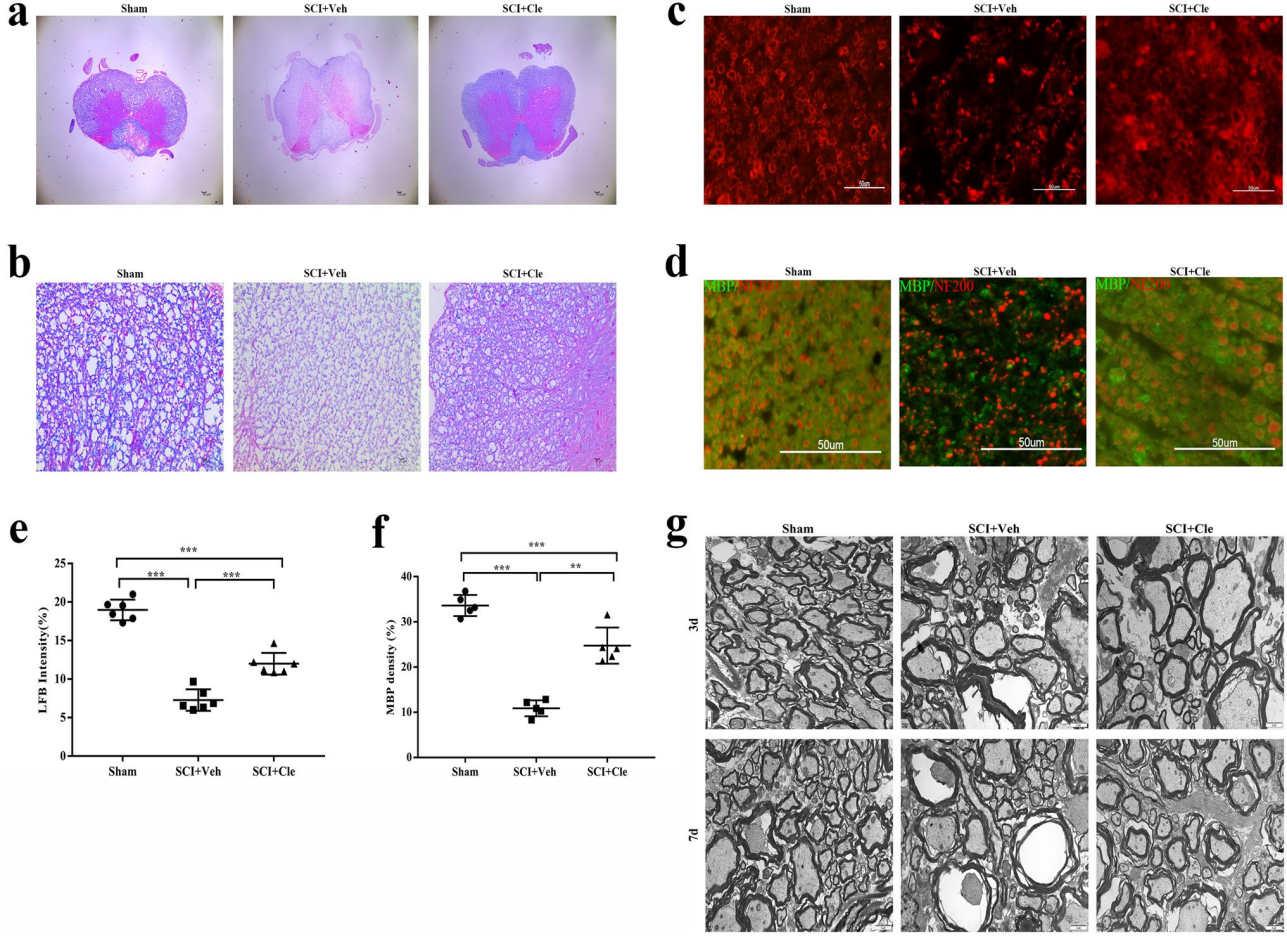
Clemastine enhances myelination in spinal cord injury. **a** Spinal cord cross-sections were stained with LFB/HE at 14 days post lesion (dpl). **b**, **e** Confocal images and quantifications of myelinated fibers in spinal white matter. Blue-green arrows point to myelin, and red arrows point to axons. Animals treated with clemastine (SCI + Cle group, n = 5) had more myelin than the vehicle-treated group (SCI + Veh group, n = 5) at 14 days after SCI. Scale bar, 10 µm. ***p < 0.001 by one-way ANOVA followed by Tukey’s post hoc test. **c** Spinal cord sections immunostained for MBP (red) showing myelin staining in each group. **d** The co-labeling of MBP (green)and NF200(red). **f** Quantification of MBP + density in the clemastine-treated group was obviously higher than that in the vehicle-treated group. Scale bar, 50 µm. *p < 0.05, **p < 0.01, and ***p < 0.001 by one-way ANOVA followed by Tukey’s post hoc test.(G) Representative electron microscopy images of myelin in the sham group (n = 5), SCI + Veh group (n = 5) and SCI + Cle group (n = 5) after3 d.p.l. and 7d.p.l. Scale bar, 2 µm

To determine whether the improved neurobehavioral recovery in clemastine-treated rats was due to changes in myelination, myelin was examined at the protein and ultrastructure levels. Myelin retention after SCI was evaluated by LFB myelin staining (Fig. 3A, B). In the vehicle group, the white matter area of the spinal cord tissue showed a lightly stained flaky demyelinating area, and the tissue structure was considerably loosened and severely damaged. Additionally, nerve fibers were disordered, and the tissue arrangement gap was significantly widened. However, in the clemastine-treated animals, the shape of the myelin sheath in the white matter area of the spinal cord tissue was relatively complete, the area was heavily stained, the demyelination phenomenon was considerably improved, and the tissue arrangement gap was smaller and tighter. A trend toward increased LFB intensity in clemastine-treated rats was detected, compared with the vehicle-treated animals (Fig. 3E). Similarly, immunostaining of MBP, which is a marker for myelin, revealed a considerably increased area of myelin in SCI rats treated with clemastine compared with the vehicle-treated group (Fig. 3C, D). The level of MBP in the clemastine-treated group was not considerably different from the sham group in the spinal cord (Fig. 3F), suggesting a delay of MBP expression defects by clemastine after SCI.

To explore whether clemastine can effectively prevent initial demyelination, transmission electron microscopy was employed to reveal the myelin status of each group on the third and seventh day of SCI. Several literature studies have shown that myelin shedding is a progressive process. The transmission electron microscopy results revealed that the myelin sheath status on the third and seventh day indicates that the myelin sheath in the clemastine treatment group and the vehicle group did not fall off, and that it is in a process of continuous degeneration (Fig. 3G). The degree of degeneration of the myelin sheath in the clemastine treatment group was lower than that in the vehicle group, which indicates that the clemastine drug has a protective effect on the myelin sheath.

The 14-day remyelination status, revealed by transmission electron microscopy (Fig. 6A) and the 14-day MBP expression level show that clemastine enhance myelination in SCI.

### Clemastine Promotes Myelination by Promoting the Differentiation of Oligodendrocytes in Spinal Cord Injury

**Fig. 4:**
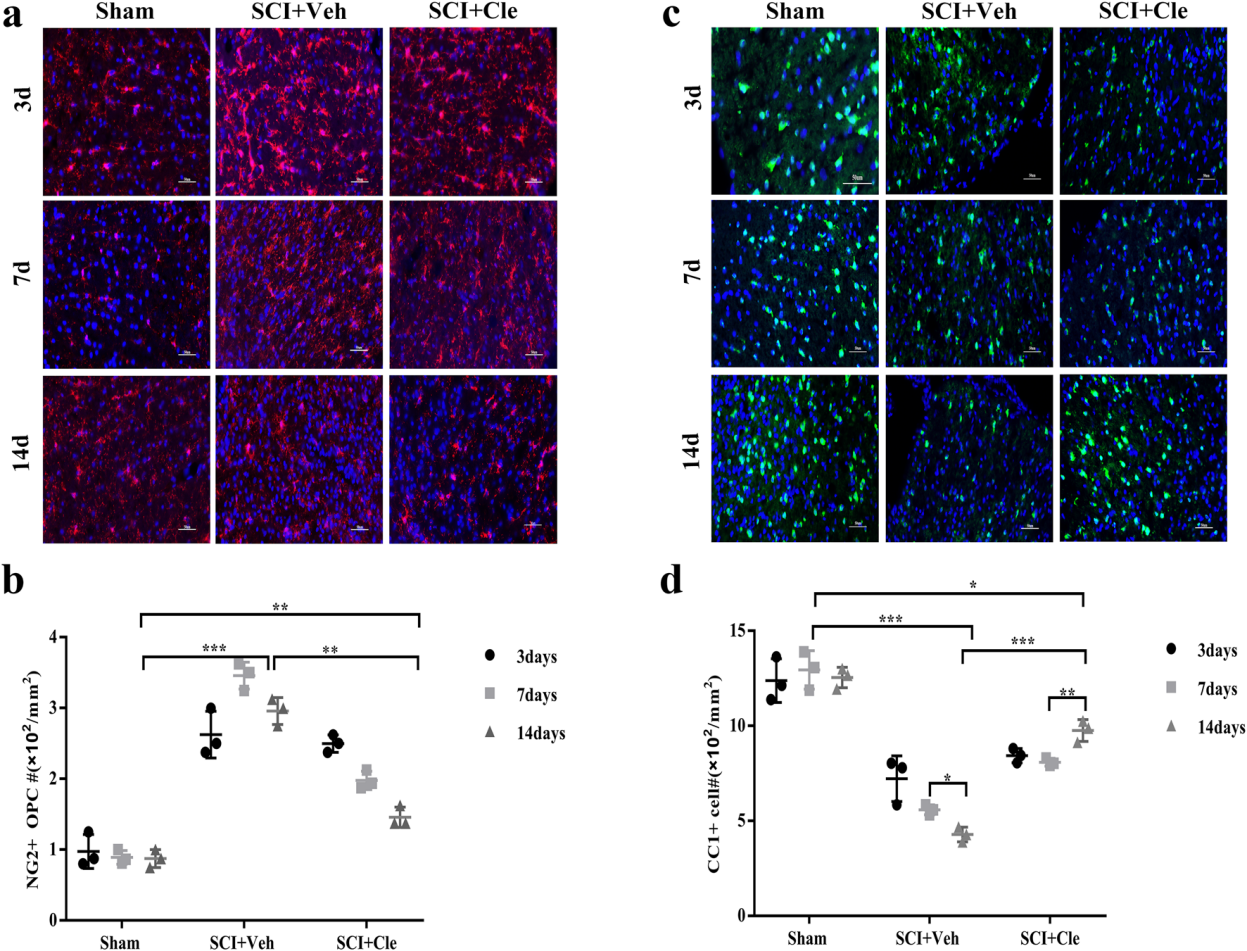
Clemastine enhances oligodendrocyte differentiation in SCI. **a** Immunostaining for NG2 (red) displays oligodendrocyte precursor cells in the spinal cord white matter tracts of the sham group, SCI + Veh group and SCI + Cle group. DAPI (blue) was used as a nuclear counterstain. Scale bar, 50 µm. **b** Quantification of NG2-positive cell numbers in the spinal cord white matter tracts. **c** Analysis of CC1 immunostaining of the spinal cord white matter tracts from the sham group, SCI + Veh group and SCI + Cle group. Scale bar, 50 µm (D) CC1-positive cell numbers in the spinal cord white matter tracts were quantified. [n = 3 for each group] *p < 0.05, **p < 0.01, and ***p < 0.001 by one-way ANOVA followed by Tukey’s post hoc test

The enhanced myelination by clemastine could be due to increased OPC proliferation or differentiation. However, enhancing myelination often fails, primarily owing to the failure of OPC lineage progression rather than a depletion of OPCs, many of which persist in chronic demyelinated lesions. To distinguish among these possibilities, OPC and mature oligodendrocyte populations in the spinal cord were analyzed by immunostaining. Immunostaining of NG2, which is a marker for OPCs, revealed a significantly increased number of progenitors in SCI rats treated with vehicle compared with the clemastine-treated groups (Fig. 4A, B). It is postulated that the enhanced myelination by clemastine results from increased OPCs differentiation. To understand the effect of clemastine on mature oligodendrocytes, we identified a significant increase in CC1 + cells, a marker for mature oligodendrocytes, in the SCI of clemastine-treated rats compared with vehicle-treated rats (Fig. 4C, D). The clemastine-treated groups showed a trend of increased cell numbers, which reached significance.

These results indicate that clemastine is effective in promoting OPC differentiation and OL formation in rat models of SCI.

### Clemastine Delays Significant Axonal Degeneration after Compressed Spinal Cord Injury in Rats

Axonal degeneration is considered one of the major mechanisms of the degeneration process and occurs in different phases of SCI. The presence of axonal degeneration after SCI has been established, and different treatments for neurologic injury have been implemented. These treatments have not been effective in most cases. The role of clemastine in axonal degeneration in SCI was investigated.

**Fig. 5:**
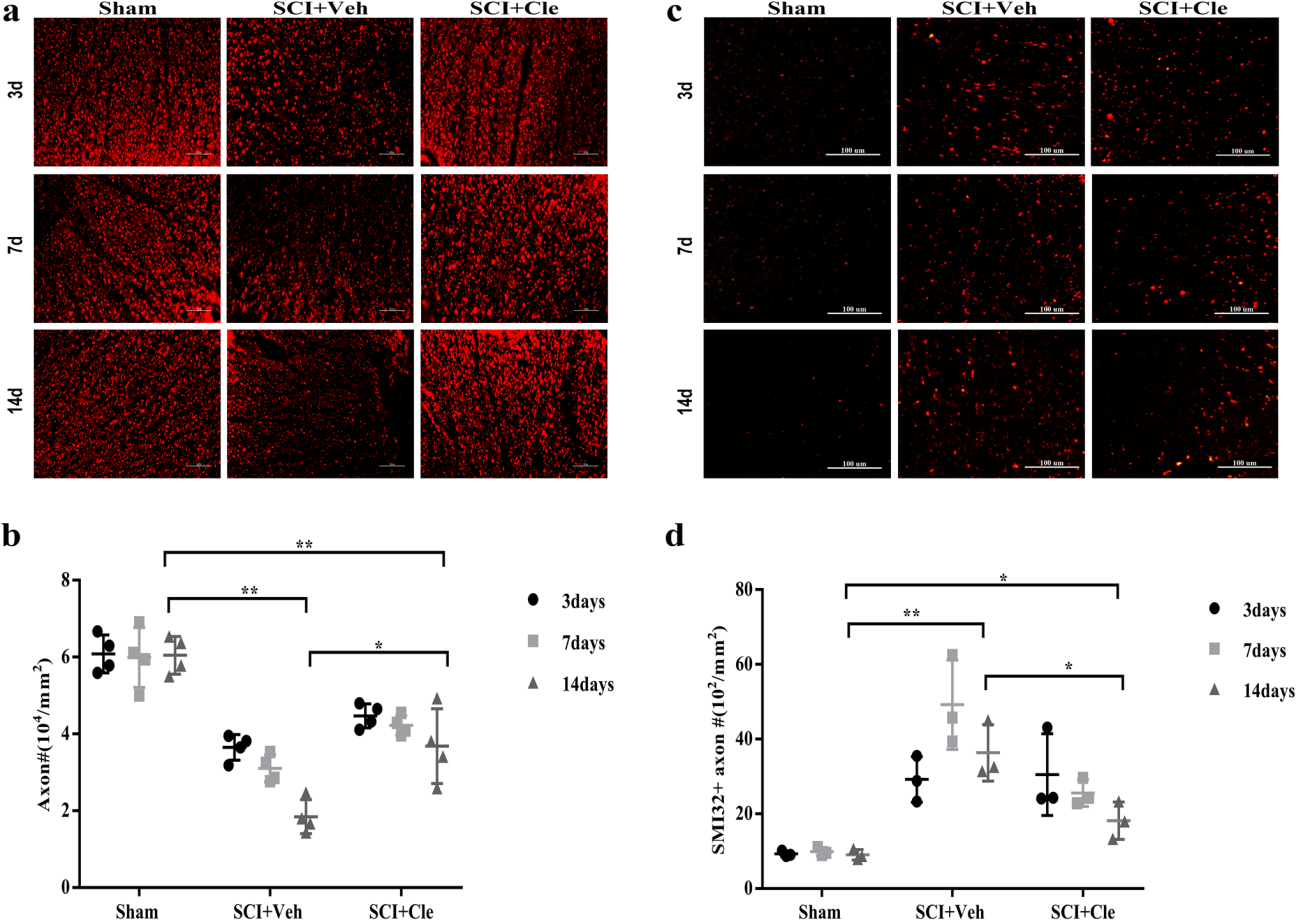
Enhanced myelination prevents axonal loss in spinal cord injury. **a** Spinal cord cross sections stained with NF200 (red) showing number of axons in the sham 、SCI + Veh and SCI + Cle groups after 3, 7 and 14 d.pl. Scale bar, 50 μm. **b** Quantification of NF200 + axon numbers from each group(n = 4). **c** Representative images showing SMI-32 (red) in the spinal cord after 3, 7 and 14 d.pl. Scale bar, 100 μm. **d** Numbers of SMI32 + axon were quantified. Error bars represent mean ± S.E.M. Significance based on Student’s t-test with the respective controls. n = 3 for each group.*p < 0.05 and **p < 0.01 by one-way ANOVA followed by Tukey’s post hoc test. Data are the mean ± SEM

To investigate this issue, the number of NF200-positive axons was measured between different groups. It is worth noting that at the same piont, the number of NF200-positive axons in the clemastine-treated group was significantly higher than the corresponding number the vehicle group, indicating that clemastine delayed the significant axon loss after SCI (Fig. 5A, B). It can be seen that the most significant time period for the difference of NF200-positive axons between the vehicle group and the drug treatment group is 14 days after SCI. In addition, SMI32 staining was also performed, because it mostly accumulates in degenerated axons. In the vehicle group and the clemastine-treated group, the granular changes of axons can be seen in the SMI32 staining, indicating that axons have undergone degeneration. The number of SMI32 stained axons and granular changes in the clemastine-treated group were less than those in the vehicle group. This result can strongly support the claim that clemastine prevents degeneration (Fig. 5C, D). This result can strongly support the claim that clemastine prevents degeneration and loss.

**Fig. 6:**
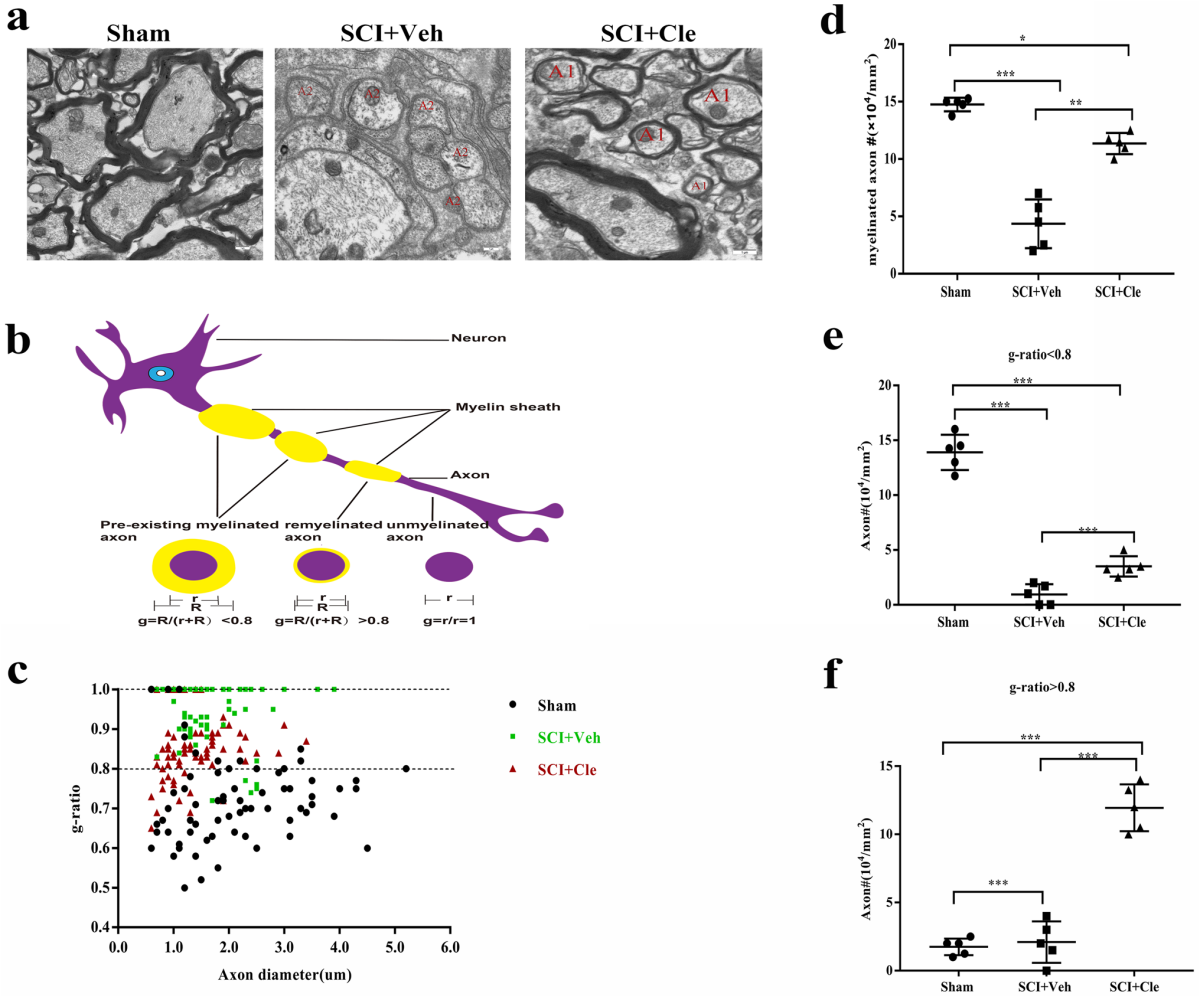
Myelin ultrastructure in sham group, SCI + Veh and SCI + Cle spinal cord. **a** Representative electron microscopy images of myelinated axons and demyelinated axons in the spinal cord white matter tracts of the sham group (n = 5), SCI + Veh group (n = 5) and SCI + Cle group (n = 5) after 14 d.p.l. **b** A schematic diagram of the pre-existing myelinated axons, remyelinated axons (A1) and demyelinated axons (A2). **c** Quantification of myelin sheath thickness and the proportion of myelinated and unmyelinated axons in SCI + Veh (green) and SCI + Cle (red). The scatterplot displays g-ratios of individual axons as a function of axonal diameter. **d** Quantification of myelinated axons from the sham group, SCI + Veh and SCI + Cle group. **e**, **f** Quantification of myelinated axons and axons with a g-ratio > 0.8(E) or < 0.8(F) in the clemastine-treated and vehicle-treated group and sham group. *p < 0.05, **p < 0.01, and ***p < 0.001 by one-way ANOVA followed by Tukey’s post hoc test. n.s., not significant. Data are the mean ± SEM

Furthermore, to determine whether myelination is neuroprotective and preserves axonal integrity in SCI models, the axons in the spinal cord ventral white matter were examined with transmission electron microscopy (Fig. 6 A). Remyelinated axons were identified by thinner myelin sheaths, as compared to the pre-existing myelinated axons with thicker myelin sheaths (Fig. 6B). demyelinated and degenerating axons were easily detected with a low number of remyelinated axons and pre-existing myelinated axons. In contrast, remyelinated axons were more consistently observed in the clemastine-treated rats (Fig. 6A). The overall myelinated axon density was significantly decreased in vehicle-treated rats, as compared to the clemastine-treated rats, suggesting that less axons undergo degeneration in the clemastine-treated rats (SCI + Cle: 11.4 ± 1.05; SCI + -Veh: 4.31 ± 2.44, p < 0.01. Figure 6D). Furthermore, the g-ratio of the axons in SCI was quantified, and the percentage of unmyelinated axons (g-ratio = 1) was decreased in the clemastine-treated spinal cords, suggesting that myelination was enhanced in the clemastine-treated rats (Fig. 6C). The majority of the axons exhibited g-ratios below 0.8 (pre-existing myelinated axons) in the sham spinal cord white matter (Fig. 6B). The pre-existing myelinated axon density (g-ratio < 0.8) significantly decreased in the clemastine-treated group and the vehicle groups (Fig. 6F), suggesting the clemastine- and vehicle-treated groups experienced a severe demyeination process. Remyelinated axon density (g-ratio > 0.8) was detected in the clemastine-treated groups and was significantly increased compared to the vehicle-treated groups (Fig. 6E), demonstrating that remyelination by clemastine is an important factor to support axonal integrity in SCI.

To sum up these results, the findings suggest that the clemastine treatment is playing a key role in preserving axon integrity after SCI.

**Fig. 7:**
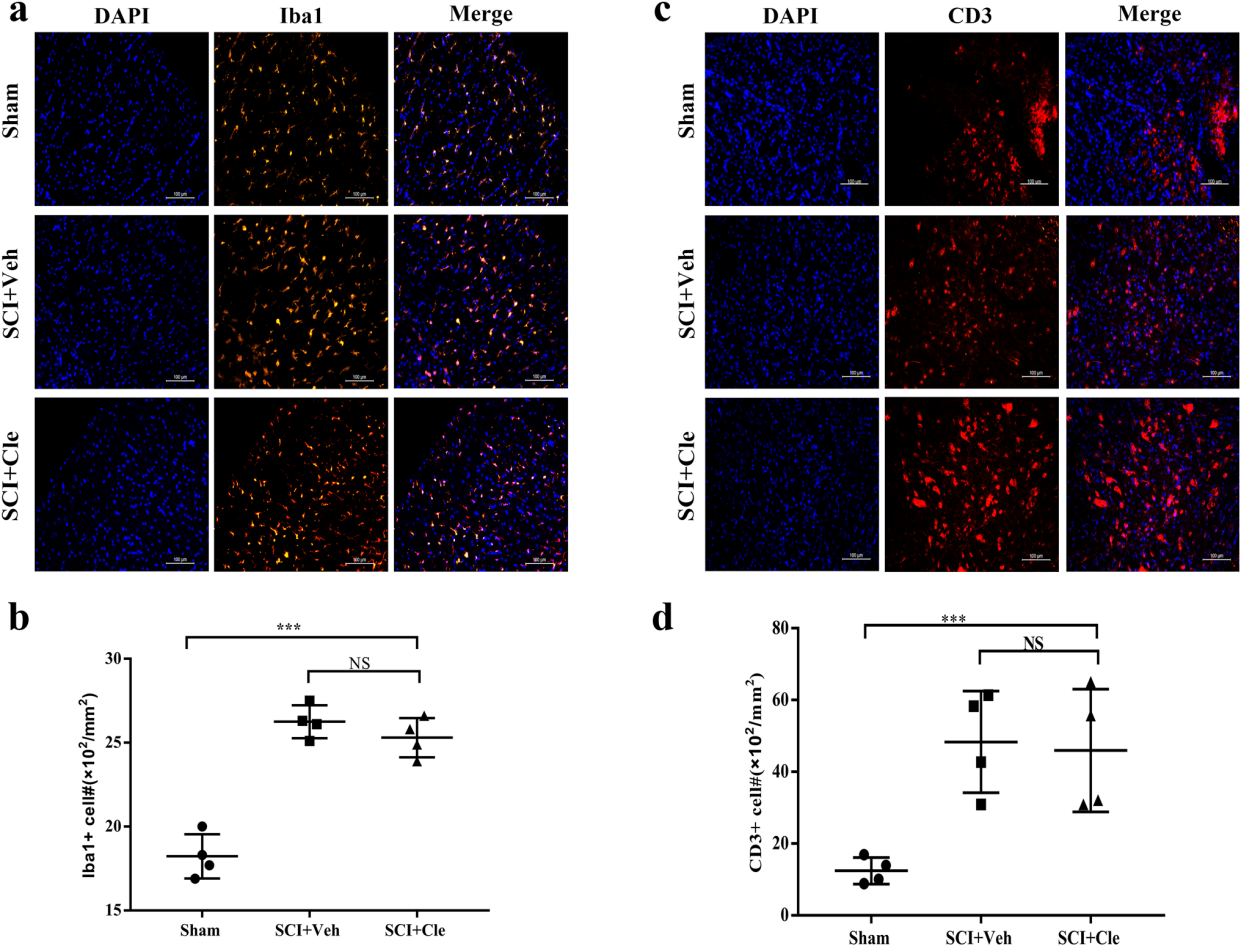
Clemastine treated and vehicle rats were analyzed for T-cells and macrophages/microglia in sSCI. **a**, **c** Sections were immunostained for CD3 (T-cells; red), and Iba1 (macrophages and microglia; red). **b**, **d** Density of CD3 and Iba1 positive cells were quantified in demyelinated lesions from vehicle and clemastine treated rats in spinal cord injury. Error bars represent mean ± S.E.M. and all experiments were performed in triplicate. *p < 0.05, **p < 0.01, and ***p < 0.001 by one-way ANOVA followed by Tukey’s post hoc test. n.s., not significant

To examine if inflammation is altered by clemastine treatment, we performed immunofluorescence staining for Iba1 to label microglia in the follow-up period (Fig. 7A.B). At the same time, CD3 immunostaining was performed to observe the changes of immune cells during treatment (Fig. 7C, D). The results indicate that neither Iba1 positive nor CD3 positive cell density was altered by clemastine treatment in the SCI lesion, as compared to vehicle groups.

## Discussion

Current treatments for SCI include traditional drug therapy [22], surgery [23], cell therapy [24], and tissue engineering [25]. Evidence suggests that the safety and effectiveness of traditional medicine and surgical treatment are controversial, and cell transplantation and material treatment are expensive and difficult to operate. This study focused on establishing a qualified animal model with high similarity to human SCI and applying safe drug therapy to the model to achieve the purpose of treating SCI.

Evidence suggests that the cessation of OPC differentiation in neurodegenerative diseases and neurological trauma is a key factor in the failure of myelin production, leading to progressive nervous system damage. White matter neuron loss, demyelination and axonal degeneration are a series of pathological changes caused by compressive SCI [26–28]. The persistence of OPC differentiation is particularly important to remyelination after SCI [29]. Clemastine belongs to a group of anti-muscarinic compounds that have been newly observed to upregulate the differentiation and remyelination of oligodendrocytes [18, 30]. The results in this study further support this claim as the amount of myelination in the SCI model with clemastine treatment was found to be greater than the vehicle group. Therefore, the use of clemastine is recommended for this purpose.

Significant demyelination after SCI leads to neurological defects, including motor coordination disorders and hindlimb paralysis [31, 32]. Through BBB scoring, sloping board and grid walking experiments, our results suggest that clemastine treatment is beneficial to the recovery of motor function in SCI. Based on these results, we postulate that the effect of clemastine on behavioral changes may be related to enhanced myelination in SCI.

The myelin sheath is a layer of fatty tissue that wraps around the axons of certain neurons. It protects the axons, provides an insulating effect and improves the conduction speed of nerve impulses. The myelin segment of all 30–80 different axons is produced and maintained by one oligodendrocyte [33, 34]. Through transmission electron microscopy, the microstructure was observed, and the images showed that after 14 days of SCI, the number of remyelinated axons in the clemastine treatment group was higher than in the vehicle group. This result indicates that clemastine could block axon loss by promoting myelination. In addition, the results of SMI32 staining showed that the proportion of axon degeneration in the clemastine treatment group was lower than in the vehicle group. This result supports that the effect of clemastine on axons may be partly due to the initial protection of SCI.

Clemastine cannot fully restore the destruction of the myelin sheath and dysfunction caused by SCI. Demyelination is not the only factor that causes dysfunction of SCI; SCI has a series of complex pathophysiological phenomena including cell death, axon collapse and demyelination, glial scar formation, inflammation and other pathological defects. In the treatment of SCI, a combination therapy is recommended to be adopted based on pathological factors to promote the complete recovery.

The results of this study show that clemastine treatment improves myelination, prevents axon degeneration and subsequently promotes functional recovery after SCI. However, the mechanism of clemastine promoting the differentiation of OPCs remains unclear. As a muscarinic receptor antagonist, clemastine can activate IL-1β through P38 in the synaptic nucleosome in hypoxic-ischemic brain injuries [35]. The MAPK/ERK pathway is a well-characterized positive regulator of oligodendrocyte differentiation and myelination [36, 37]. The G protein-coupled receptor signaling pathway involved in promoting the differentiation of oligodendrocytes has not been fully elucidated. Therefore, future experiments will explore the hypothesis that clemastine promotes myelination through the ERK pathway mediated by G protein-coupled receptors in demyelinating diseases.

In summary, clemastine treatment of SCI was confirmed to enhance oligodendrocyte differentiation and myelination, thus helping to delay axonal degeneration and promote the recovery of motor function. Therefore, our findings provide new insights for understanding the role of myelination and oligodendrocyte function in improving the recovery of nerve function, and can help create a new perspective to treat and examine SCI.

## Data Availability

The data that support the findings of this study are available from the corresponding author upon reasonable request.

## Declarations

### Research Involving Human and Animal Participants

The study was approved by the Laboratory Animal Center of Chongqing Medicine University in China (license No. SYXK (Su) 2018–0003).
